# High-Quality Draft Genome Sequence of Low-pH-Active *Veillonella parvula* Strain SHI-1, Isolated from Human Saliva within an *In Vitro* Oral Biofilm Model

**DOI:** 10.1128/genomeA.01684-15

**Published:** 2016-02-18

**Authors:** Anna Edlund, Quanhui Liu, Michael Watling, Thao T. To, Roger E. Bumgarner, Xuesong He, Wenyuan Shi, Jeffrey S. McLean

**Affiliations:** a Department of Periodontics, University of Washington, Seattle, Washington, USA; b Microbial and Environmental Genomics, J. Craig Venter Institute, La Jolla, California, USA; c School of Dentistry, University of California, Los Angeles, Los Angeles, California, USA; d Department of Microbiology, University of Washington, Seattle, Washington, USA

## Abstract

We announce here a draft genome sequence of *Veillonella parvula* strain SHI-1, obtained from healthy human saliva, discovered to be active at low pH using metatranscriptomics within an *in vitro* oral biofilm model. The genome is composed of 7 contigs, for a total of 2,200,064 bp.

## GENOME ANNOUNCEMENT

Oral bacteria belonging to the genus *Veillonella* are common inhabitants of biofilms on tooth surfaces, the tongue, and the buccal mucosa (1–3). *Veillonella* interacts with a broad group of bacterial taxa and is proposed to promote the growth of both health- and disease-associated bacteria (4–7). In-depth knowledge of these interactions at the gene and molecular levels is lacking. However, in one of our earlier studies of an *in vitro* biofilm community of >100 bacterial species, a previously uncultivated *Veillonella parvula* community member increased in abundance and gene transcription activity at low pH (pH 4.2 to 5.2) in response to sugar amendment (8). *V. parvula* was also previously identified and isolated from patients with chronic periodontitis (2) and severe early childhood caries (9). Here, we report the draft genome sequence of *V. parvula* strain SHI-1, isolated from a diverse *in vitro* biofilm derived from the saliva from a healthy patient (8). The *V. parvula* SHI-1 isolate and genome sequence obtained here will aid in future analysis and annotation efforts of metagenomic and transcriptomic data from the human oral microbiome.

Samples for *Veillonella* isolation were collected 9 h after glucose amendment of the *in vitro* biofilms, grown as described previously (8, 10). Selective agar plates for *Veillonella* were prepared, as described in Egland et al. (11), from Todd-Hewitt broth (THB) supplemented with 0.6% lactic acid. Plates were prepared anaerobically, and 20 µl of resuspended biofilm sample was spread on each plate and incubated anaerobically at 37°C until colonies appeared. Individual colonies were grown in liquid THB and lactic acid under the same incubation conditions described above (without shaking) prior to DNA extraction. DNA was isolated, as described by McLean et al. (12), using the DNeasy blood and tissue kit (Qiagen, Inc., MD) and eluted in a final volume of 200 µl of water. Taxonomic identity and culture purity were determined by partial 16S rRNA gene sequencing by Eurofin Genomics (Huntsville, AL), using forward primer 341F (CCTACGGGAGGCAGCAG) (13).

A paired-end Illumina library was prepared from a DNA extract of a 16S-validated growth culture and sequenced on a MiSeq sequencer (Illumina, Inc.) (2 × 300 bp). All quality-trimmed reads were *de novo* assembled using SPAdes version 3.61 (14, 15). The contigs were inspected for k-mer frequency consistency, and 7 scaffolds were used for further analyses.

The draft genome is 2.2 Mb, with an overall G+C content of 38.7%. Gene annotation using the Prokaryotic Genome Automatic Annotation Pipeline (PGAAP), provided by the National Center for Biotechnology Information (NCBI), identified a total of 2,055 genes, consisting of 2,005 coding sequences, 45 tRNAs, one small subunit (SSU) 16S rRNA, one large subunit (LSU) 23S rRNA, and 3 SSU 5S rRNAs. The average nucleotide identity (ANI) (16) between SHI-1 and its closest phylogenetic neighbor, ATCC 17748, is 96.51%.

### Nucleotide sequence accession numbers.

This whole-genome shotgun project has been deposited at DDBJ/EMBL/GenBank under the accession no. LOJO00000000. The version described in this paper is version LOJO01000000.
